# Self-Organisation in Spatial Systems—From Fractal Chaos to Regular Patterns and Vice Versa

**DOI:** 10.1371/journal.pone.0136248

**Published:** 2015-09-24

**Authors:** Michal Banaszak, Michal Dziecielski, Peter Nijkamp, Waldemar Ratajczak

**Affiliations:** 1 Faculty of Physics, A. Mickiewicz University, ul. Umultowska 85, 61-614 Poznan, Poland; 2 Faculty of Geography and Earth Sciences, A. Mickiewicz University, ul. Dziegielowa 27, 61-680 Poznan, Poland; 3 Faculty of Economics and Business Administration, VU University, De Boelelaan 1105, 1081 HV Amsterdam, The Netherlands; UNIVERSITY OF LAUSANNE, SWITZERLAND

## Abstract

This study offers a new perspective on the evolutionary patterns of cities or urban agglomerations. Such developments can range from chaotic to fully ordered. We demonstrate that in a dynamic space of interactive human behaviour cities produce a wealth of gravitational attractors whose size and shape depend on the resistance of space emerging inter alia from transport friction costs. This finding offers original insights into the complex evolution of spatial systems and appears to be consistent with the principles of central place theory known from the spatial sciences and geography. Our approach is dynamic in nature and forms a generalisation of hierarchical principles in geographic space.

## Introduction

The chief goal of this paper is to demonstrate that social-spatial interactions may generate a wealth of phase transitions varying from chaotic patterns to organised structures, even in geographic spaces endowed with robust spatial-order features. Attempts to identify spatial order through hexagonal networks embracing settlement units of various sizes have been made in the spatial sciences for a long period, witness the pioneering work of Reynaud [1]. Christaller [2] was the first to conceptualize spatial order as a so-called hierarchical hexagonal network, framed as central place theory (CPT). This spatial architecture was later on cast in a deductive economic modelling framework by Lösch [3], while it was generalized to global market places by Tinbergen [4]. The CPT has induced numerous empirical and conceptual studies ever since. The statistical test of such hierarchical orders in space has prompted an avalanche of studies centred around the so-called Zipf‘s Law (for a review, see [5]). CPT has become an established analysis framework [6, 7], that has also been invoked by many other authors outside economics and geography, including Prigogine and Stengers [8, 9]. This paper corroborates the validity of the CPT approach through the discovery and analysis of a strange gravitational attractor in dynamic space.

## Central Places in a Dynamic Perspective

**Fig 1:**
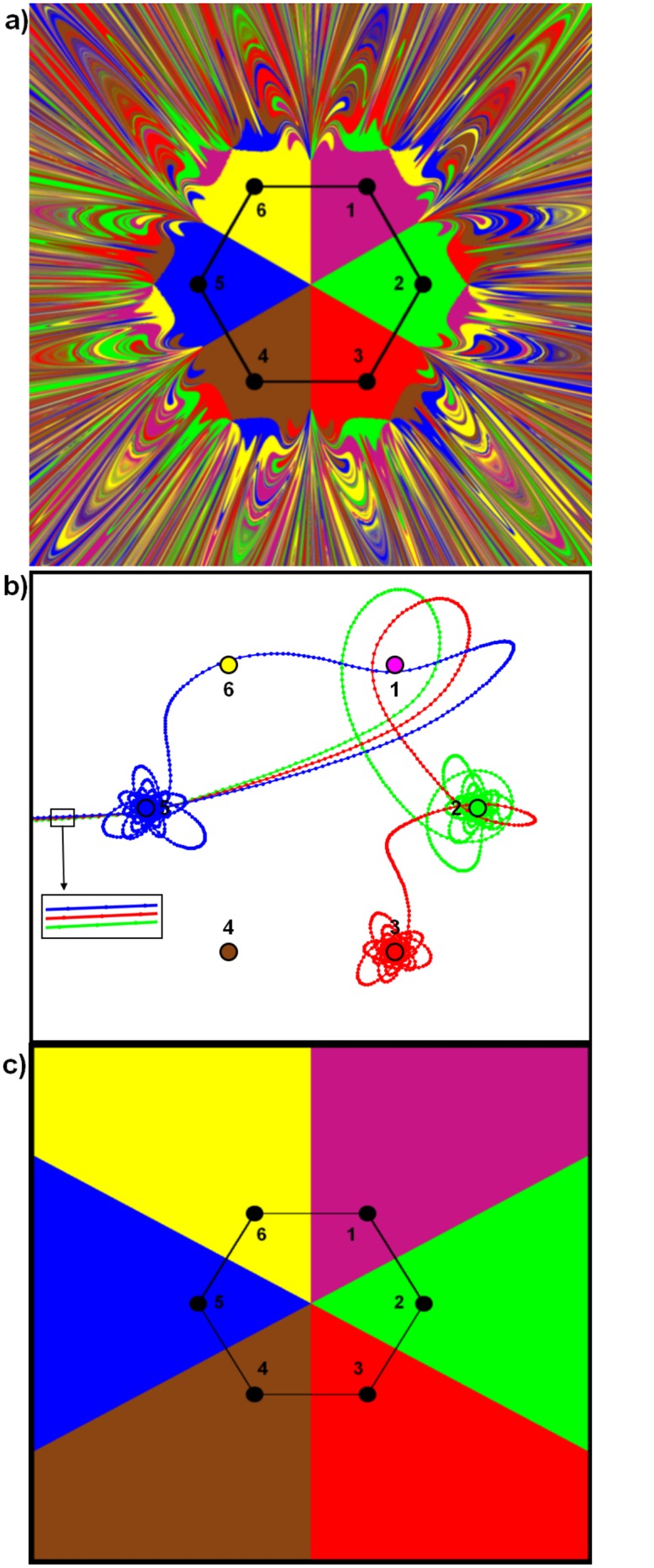
a) Chaos and order in a hexagon with cities of equal masses, with the following parameters: *μ* = 0.09, *m* = 1, *k* = 1, and *h* = 0.02, which will be described below. Cities, indicated by black circles, have the following coordinates: $\left(\sqrt{3},1.0\right)$, (0.0,2.0), $\left(-\sqrt{3},1.0\right)$, $\left(\sqrt{3},-1.0\right)$, (0.0, −2.0),and $\left(-\sqrt{3},-1.0\right)$. The distance between neighbouring cities is 2; b) three trajectories; c) transition to order in hexagonally-placed cities with *μ* = 0.7.

Our study will present the equations of motion (see Eq 10) for simulating an ideal-typical hierarchical spatial system of places or settlements. The simulation experiment presented below is based on the theory of geographical potential, which is an important component of social physics that has been applied in socio-economic geography for more than 100 years (starting with [10], [11, 12] and [13], and more recently [14, 15]). The premise of our study is that humans are ‘thinking particles’ in geographical space, who interact on the basis of economic, social or cognitive proximity. The behavioural foundation and justification of the concept of ‘social physics’ can be found inter alia in [16], where the authors provide a clear methodological underpinning for a behavioural conceptualisation micro processes in relation to macro outcomes. Clearly, human systems are not identical to statistical mechanics systems, but their dynamic behaviour in geographic space may exhibit similar movements. In accordance with the idea of geographical potential, our experiment rests on the assumption that settlement units, e.g. towns and cities, produce attraction areas, or gravitational attractors for agents. This means that an arbitrary, uninformed agent who enters the attraction space spanned by various towns tends to take first a chaotic path, before finally being attracted by one of the towns. It will ultimately find itself in its gravitational attractor. This situation is illustrated in Fig 1a which depicts six towns in a hexagonal CPT pattern. For numerical calculations, we use a 2-dimensional grid. The x and y coordinates are set from -5 to 5, so that we have a 10 by 10 field. The starting points of agents are located at equal increments of 1/700. So we have 7,000 points on the x axis and 7,000 points on the y axis, which gives a total of 49 million starting points. If the agent starts from point (-5,5) and stops on a hexagon point, for example (0,2) labelled green, then this starting point will also be labelled green. This procedure is repeated for every starting point with a corresponding colour.

In the simulation process, the transport cost parameter *μ* is kept changing continuously. The final result—spatial order by creating gravitational attractors—was established at *μ* = 0.7. This value is more than seven times higher than *μ* = 0.09. The following formulas express the relationship between parameters *μ*_*c*_ (critical), h, m and k:

$$h=-km/U_{\left(0\right)},$$ (1)

$$\mu_{c}∼\sqrt{\frac{\bar{m}}{L}},$$ (2)

$$L=\sqrt{\frac{1}{M}\sum_{i=1}^{M}m_{i}{\left({\vec{r}}_{i}^{\;0}-{\vec{r}}_{M}\right)}^{2}}.$$ (3)

We obtained from the simulations *μ*_*c*_ = 0.5.

The agent starts from the extreme upper (NW) square and proceeds through successive, neighbouring squares arranged in layers. In this case, it is attracted by six towns. The field from which it started is marked by the colour of the town that attracted it. The procedure stops when the agent has reached the square in the lower right corner (SE).

How should Fig 1a be interpreted? It is a picture of spatial CPT order and spatial chaos. The order is represented by the area inside the hexagon which the simulation process has divided into six equal sectors. They are gravitational attractors of each of the six towns considered. The external margins of the sectors are not straight lines, because they border on chaos. Chaos is depicted by a system of alternating varicoloured layers forming highly complicated patterns. Each two layers are always evidently separated by one of a different colour. This sequence of a variety of layers appears at each magnification level and tends to infinity. This is clearly an example of pure chaos, because it is impossible to predict the place the agent will ultimately reach. The slightest change in location brings about a totally unpredictable effect. Still, in spite of their lack of regularity, chaotic dynamic systems satisfy deterministic equations of motion. That’s why the chaos depicted in Fig 1a is of a deterministic type. Understood in this way, chaos finds an exhaustive explanation in Cantor’s set (1845–1918)—the oldest deterministic fractal [17] constructed in 1883.

Fig 1 is clearly a fractal. Its geometrical structure is composed of a set of alternating coloured layers (it should be noted that two colours are always separated by another colour). Its geometrical structure is explained by the concept of Cantor’s Set, which was introduced in 1883 [18]. We may add that, among others, Saturn’s rings are also fractal [19].

Fig 1b presents three trajectories (as described by Eq 10) of an agent which starts moving across one of the three adjacent squares. The blue trajectory first goes to town 5, then to towns 6 and 1, and finally back to town 5. That is why its colour is blue. The red trajectory starts in the next adjacent square (in relation to the blue trajectory). It also first goes to town 5, but then to towns 1 and 2, to be ultimately attracted by the distant town 3 (red). The third, green, trajectory again goes to town 5, then circles around town 1 and is attracted to town 2 (green) positioned between towns 1 and 3. Fig 1b confirms the earlier conclusion that it is impossible to anticipate which town will attract certain agent if it starts from a position even slightly different from an earlier one. The agent’s movement depicts deterministic chaos. The situation inside the hexagon appears to be particularly interesting. Its area has been divided in a natural way into six equal sectors corresponding to equal forces of attraction of the six towns. The sectors converge in the hexagon’s centre, which indicates that there should be one more, seventh, town located there. This is an effect also foreseen by CPT, namely when all three underlying organizational and basic CPT rules, i.e. market, transport and administrative, are accounted for. The basic, initial pattern depicted in Fig 1a will next be modified, enter alia to identify regularities occurring in a real geographical landscape. A further analysis of the essence of these modifications will be made after the presentation of the mathematical basis of our initial pedagogical numerical experiment.

It is worthwhile reiterating that the main point of this paper is to demonstrate that agents behave in a seemingly unpredictable manner when the transport cost (friction) is low. This means that a city can successfully attract very distant agents in low-friction systems. It is practically impossible to guess which city is to attract the agent without performing detailed numerical calculations, because even a slight displacement of the initial position of the agent can change its final destination. This is generally known as deterministic chaos, and often referred to as the “butterfly effect”.

## Model

Since the pioneering works of [20, 21] and [14], it has been widely recognised that the attraction forces leading to an agglomeration can be derived from a gravitational-like potential, which can be expressed as follows:

$$U\left(r\right)=-k\frac{m}{r},$$ (4)

where *r* is the distance from the metropolitan centre, *m* is its population (also referred to as mass), and *k* is an empirical constant which depends on a variety of system-specific details, such as, for example, objects being attracted (people or goods, or maybe both). The singularity at *r* = 0 can be easily eliminated by the regularization, which introduces an additional lengthscale, *h*:

$$U\left(r\right)=-k\frac{m}{\sqrt{r^{2}+h^{2}}},$$ (5)

which not only eliminates infinity at *r* = 0, but also takes into account the basic observation that cities are not mathematical points: they have a size. The attractive metropolitan force, which is a vector, can be calculated by the standard formula known from classical mechanics:

$$\vec{F}\left(\vec{r}\right)\equiv -\vec{\nabla }U\left(r\right)=-k\frac{m}{r^{2}+h^{2}}\frac{\vec{r}}{\sqrt{r^{2}+h^{2}}},$$ (6)

where $\vec{r}\equiv \left(x,y\right)$ is a 2-dimensional vector, since the towns in our spatial system are placed on a 2D plane. As in physics, the potentials are additive scalars, and therefore can be used for systems with *M* metropolitan centres, yielding the following expression:

$$U=-k\sum_{i=1}^{M}\frac{m_{i}}{\sqrt{r_{i}^{2}+h^{2}}},$$ (7)

where *m*_*i*_ and *r*_*i*_ are the population and the distance from the *i*th city, respectively. Similarly, the attractive force can be written as:

$$\vec{F}=-k\sum_{i=1}^{M}\frac{m_{i}}{r_{i}^{2}+h^{2}}\frac{{\vec{r}}_{i}}{\sqrt{r_{i}^{2}+h^{2}}}.$$ (8)

**Fig 2:**
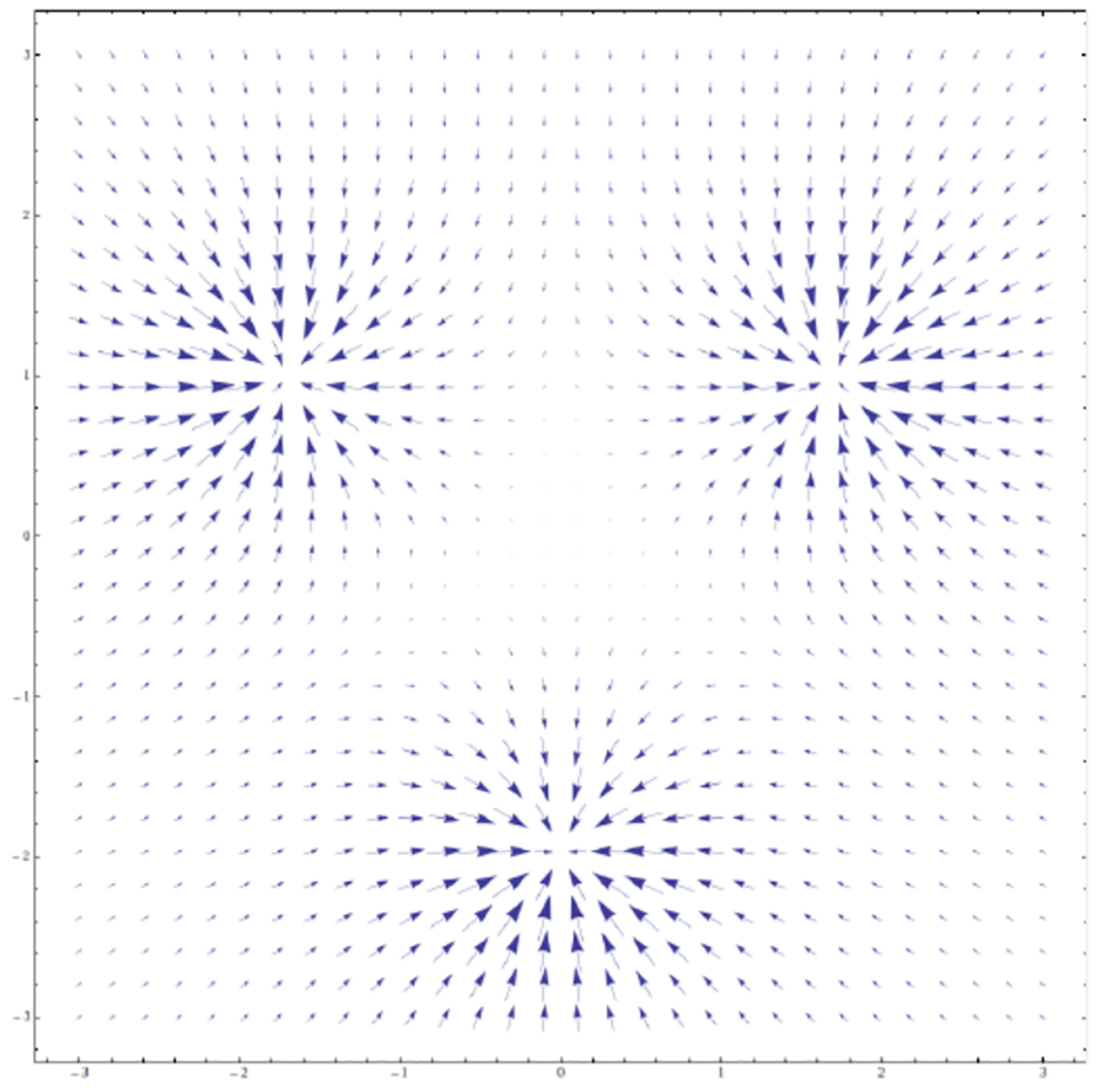
Force field for a metropolitan system with *M* = 3 and with an equal population for each city (see also [21]).

As an example, we show in Fig 2 the force field calculated from Eq (8), for a metropolitan system with *M* = 3 and an equal population for each city.

**Fig 3:**
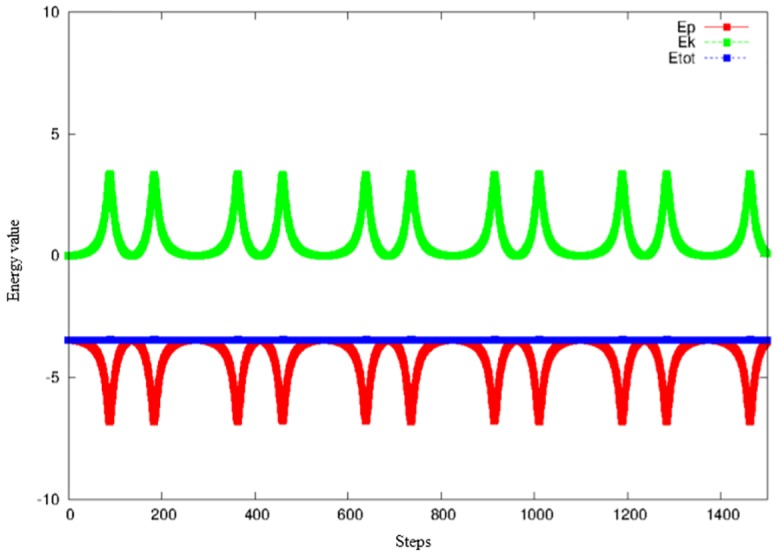
Energy conservation for *μ* = 0, Δ*t* = 0.005.

The Newtonian dynamics generated from Eq (8) does not lead to an urban centre, since the model system is energy-conserving (as in physics, energy is the sum of kinetic and potential energies, $E=\frac{1}{2}m_{A}{\dot{r}}^{2}+U$) and will be in eternal motion (see Fig 3).

The equations of motion are given below:

$$m_{A}\ddot{\vec{r}}=-k\sum_{i=1}^{M}\frac{m_{i}}{r_{i}^{2}+h^{2}}\frac{{\vec{r}}_{i}}{\sqrt{r_{i}^{2}+h^{2}}}.$$ (9)

In order to make the model more realistic we have to recognise that motion towards an attractive metropolitan centre requires some loss of energy referred to as the transport (or friction) cost [14]. While there are many mathematical models that describe those costs, it is generally accepted that a longer path will be more expensive than a shorter one. In classical mechanics the energy can be dissipated via frictional forces, which are proportional to velocity. This provides us with the inspiration to model the transport costs by frictional forces which can be added to Eq (9) as follows:

$$\ddot{\vec{r}}=-\mu \dot{\vec{r}}-k\sum_{i=1}^{M}\frac{m_{i}}{r_{i}^{2}+h^{2}}\frac{{\vec{r}}_{i}}{\sqrt{r_{i}^{2}+h^{2}}},$$ (10)

where *μ* is the friction coefficient and *m*_*A*_ = 1 without loss of generality. The friction coefficient in the behavioural parameter in our model is related to the principle of least effort of agents in dynamic space. For each starting point ${\vec{r}}_{0}=\left(x_{0},y_{0}\right)$ we can follow its trajectory by solving the above Eq (10) of motion with the initial velocity set to zero (${\vec{v}}_{0}=\left(0,0\right)$). We use the Beeman predictor-corrector algorithm [22] to solve the equation of motion. It is useful to investigate the energy landscape expressed by Eq (7) and to check the *μ* = 0 condition (see Fig 3 and ref. [23]).

In this algorithm, ‘steps’ are time steps of the numerical integration of the equation of motion. At every time step the agent changes his position, and in each new position energy (potential, kinetic and total) is calculated. The closer to the hexagon point (city), the lower the agent’s potential energy. Potential energy (red line) is calculated using Eq 7. Kinetic energy (green line) is calculated using the equation $Ek=\frac{1}{2}mv^{2}.$ Total energy (blue line) is the sum of kinetic and potential energies. Total energy does not change in this situation, so the rule of energy conservation is met. In this figure, the agent starts from point (3,3).

**Fig 4:**
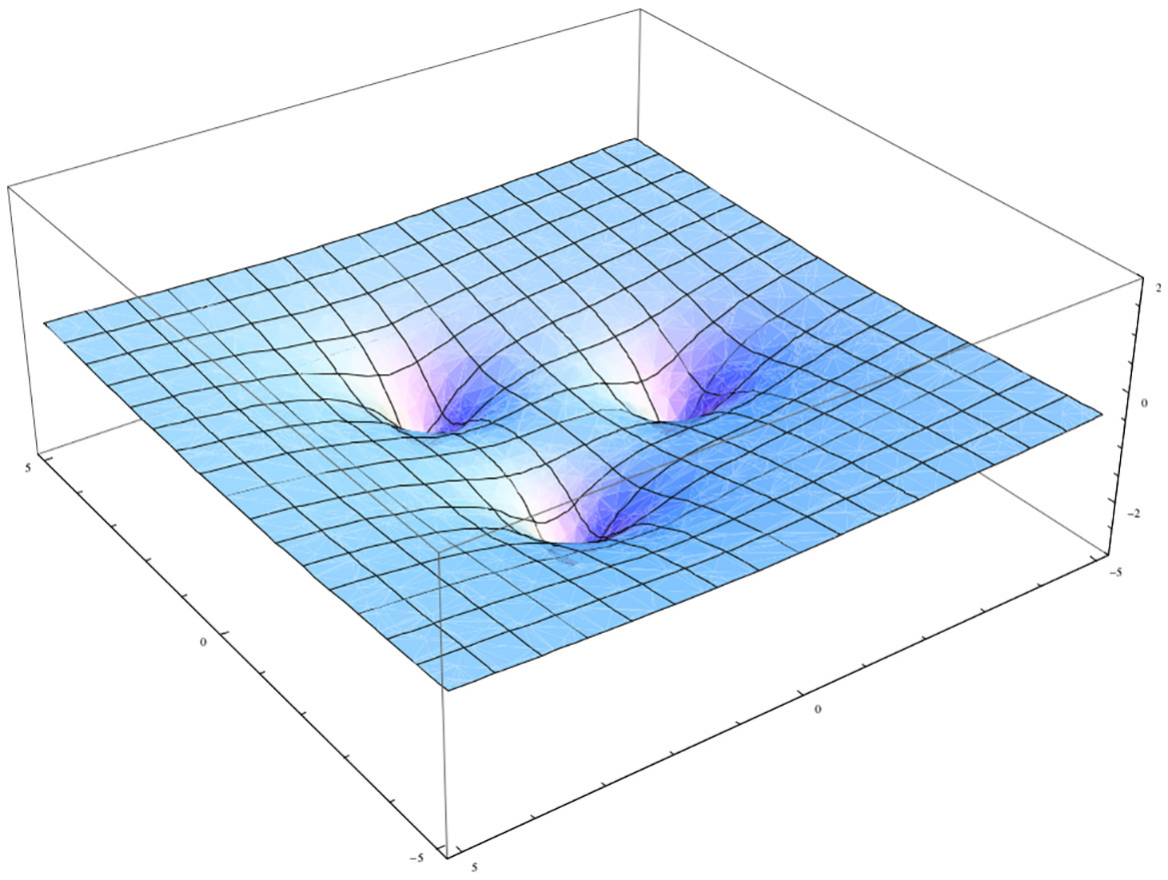
Energy landscape for a metropolitan system with M = 3 and with the same population for each city.

**Fig 5:**
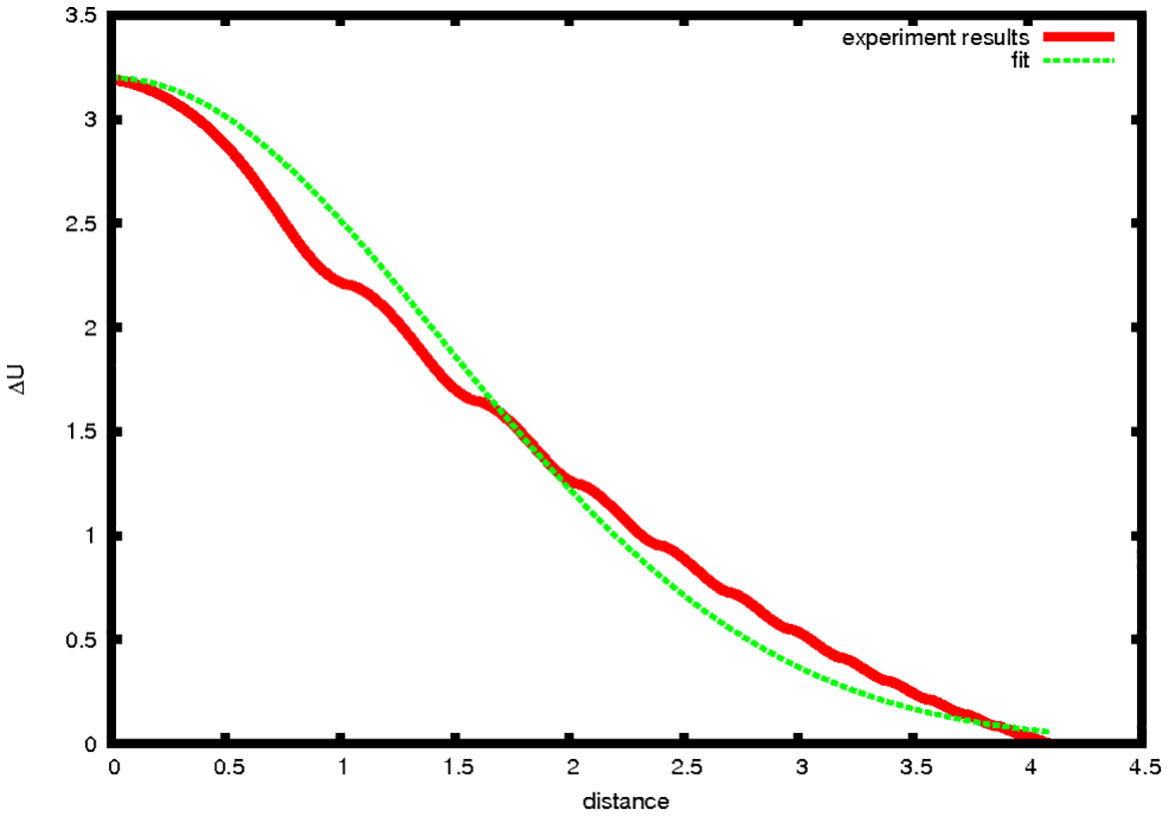
Dissipation of energy as a function of distance travelled, *s*, by an agent starting at point (1,1). Fit: 3.2 exp(−0.24*s* ^2^). Data for the red line were obtained from the numerical solution of the equation of motion Eq 10.

In Fig 4 we show the potential for a symmetric tri-city system. It is evident that every trajectory has to end in one of the metropolitan centres. We will next confirm this prediction by performing extensive numerical simulations for a wide variety of parameters characterizing the metropolitan system concerned. The structure of Model (10) allows investigating the effect of the resistance of space on the organisational level of a spatial system by changing the value of the parameter *μ*. This can be interpreted as the unit transport cost prevailing in this system. The system of attractors presented in Fig 1a stabilises when the value of *μ* changes from 0.09 to 0.7. This is corroborated by Fig 1c. The pattern it depicts accords with the effect foreseen by CPT. In other words, it is possible for a spatial system to pass from chaotic impacts to fully ordered ones, and vice versa. Fig 1c inspires us to ask an important cognitive question: what is the form of the model describing the dependence between energy loss by the agent and the distance it has travelled? Apparently, complex spatial systems can move to ordered systems under conditions of low distance costs. This intriguing question will be addressed shortly. Fig 5 presents total energy as a function of the distance (red line) of the agent. We can see that, with increasing time, the agent’s total energy decreases. This is a result of the action of friction forces (*μ* = 0.7). This behaviour differs significantly from the situation presented in Fig 3, which ignores friction (*μ* = 0).

The model in Fig 5 is a modified exponential model:

$$E_{d}=E_{p}exp\left(-\tau d^{2}\right),$$ (11)

and is written in concrete form as:

$$E_{d}=3.2exp\left(-0.24d^{2}\right),$$ (12)

where: *E*_*d*_ is the dissipated energy, *E*_*p*_ is the initial energy, *d* is the distance travelled, and *τ* is a constant energy dissipation rate. From an economic perspective, Eq (11) corresponds to the so-called rounded iceberg (Samuelson) model, referred to by Krugman and other authors in many works (Krugman [24, 25] and Fujita et al. [26]). The rounded iceberg model has been corroborated by a number of simulations concerning the remaining 5 cities of the hexagon performed by the present authors. This kind of relationship is also observed in real irregular settlement networks. Model (11) provides an interesting result, because the classical (non-modified) iceberg model has recently been subjected to critical analysis among others by McCann [27] as well as McCann and Fingleton [28].

## Self-Organisation in Real Spatial Systems

As shown above, a significant factor affecting the ordering of spatial interactions is transport costs. At minimum or zero costs, there is a practically unlimited freedom of movement in space (‘the flying carpet’ phenomenon with zero friction costs—see Rietveld [29]). Hence the movement can be chaotic, in the sense of deterministic chaos. We will now present a few real-world illustrations of hierarchical settlement patterns, one from the Netherlands and another one from the US.

**Fig 6:**
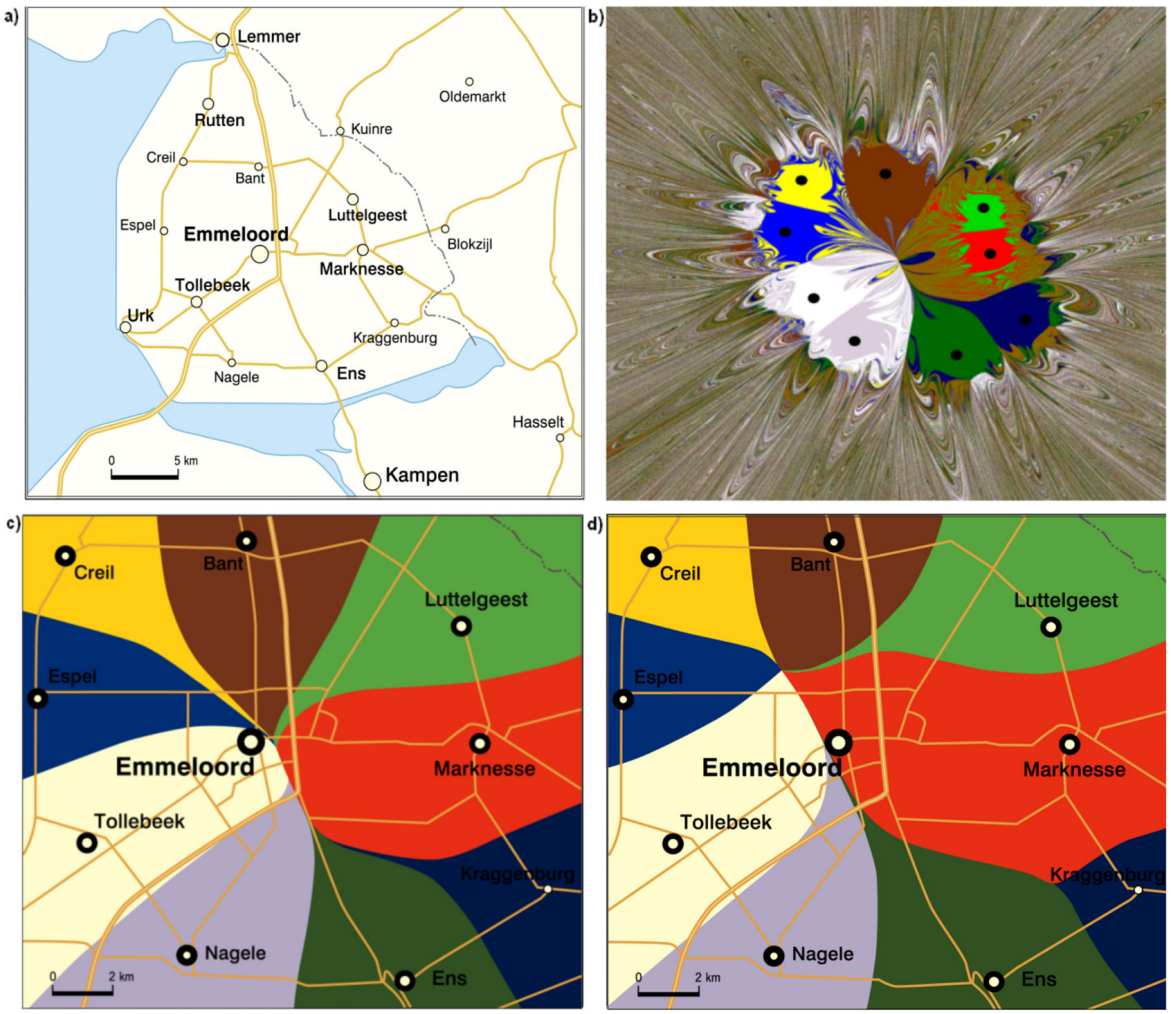
a) CPT-derived pattern of towns in the Noord Oost Polder. Source: own preparation; b) chaotic pattern at *μ* = 0.03; c) order at equal town masses (*μ* = 0.7); d) order at unequal town masses (*μ* = 0.7).

Fig 6a shows a real pattern of towns in a Dutch polder (named Noord Oost Polder) reclaimed from the sea and deliberately designed using central place theory (see [30] and [31]). This polder was planned in the 1930s, using the concept of CPT. The design rested on the assumption that the towns lying at the vertices of the figure would have 2,000 inhabitants each, and the central town—Emmeloord—10,000. The regular hexagonal pattern was only disturbed by the capricious shape on the right hand side forming the main land. The distances between these towns were small, based on convenient cycling distances.

Fig 6b now displays the effects of attraction of the towns on the assumption that they have equal masses and that transport costs are very low, viz. at *μ* = 0.03. This figure is a picture of the domination of chaos. In turn, Fig 6c and 6d present gravitational attractors produced by each of the towns (except the central one). In Fig 6c the attractors have a common point located in the centre, while in Fig 6d the common point is shifted NW in relation to the central city. This is so because the towns have unequal population numbers, different from the planned ones, which means that the structure of gravitational attractors reveals the designed pattern to have an advantage over the real one. At the same time, Fig 6d illustrates two important spatial properties of gravitational attractors: (1) the border lines of the attractors divide the sides connecting the individual towns of the system exactly in half, irrespective of whether the towns have the same or different weights, and (2) the irregularity of the pattern made up by the towns (in this case, a nonagon) and the differences in their masses affect the shape and area of their attractors. For instance, the village Kraggenburg is located at the furthest distance from the central city, while its two nearest neighbours: Marknesse and Ens, which have greater masses, at 3,847 and 3,129, respectively. That is why its attractor is partly taken over by those two towns, because the towns compete for their own areas of influence. The situation is similar in the case of Espel.

**Fig 7:**
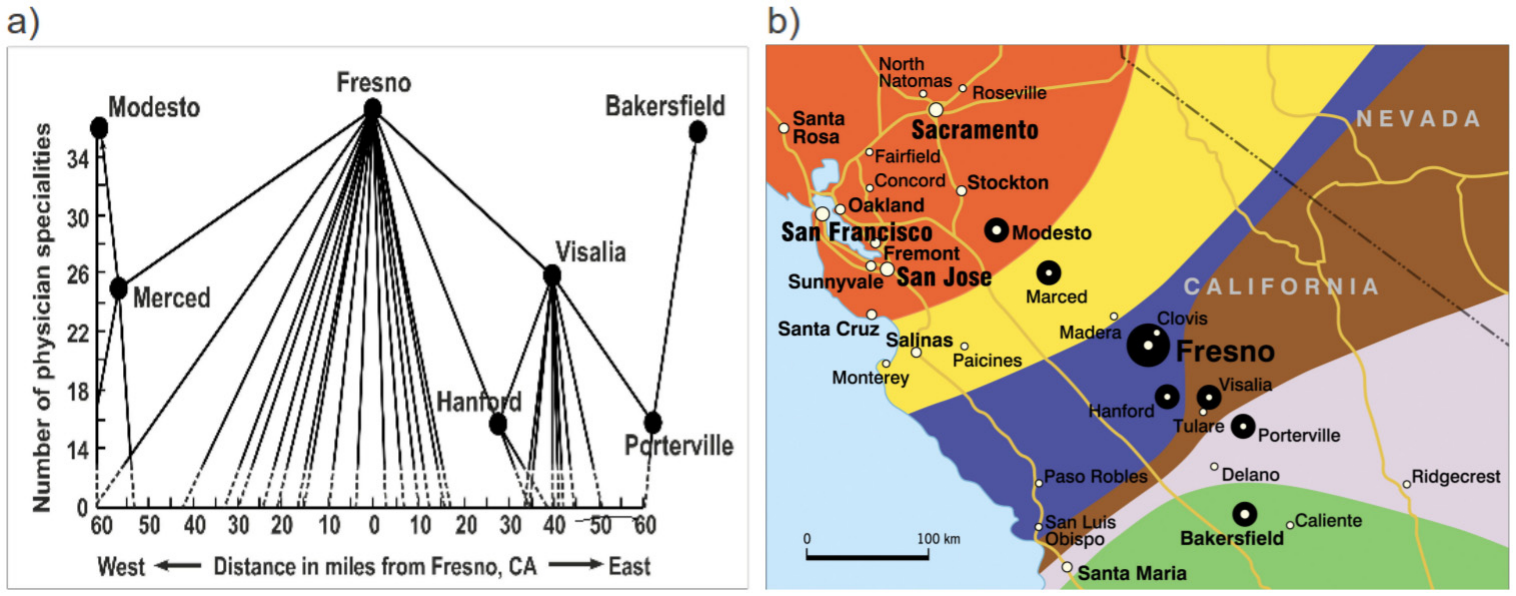
a) Referral diagram of the Fresno medical care region [32] and b) corresponding gravitational attractors of the same cities in Fresno region with different number of physician specialties works (*μ* = 3.0).

Next, we present an empirical illustration from the US. Fig 7 is inspired by a study by Smith [32], who employs CPT to delimit medical care regions, medical trade areas, and hospital service areas.

Fig 7a presents part of a hierarchical dendrogram of central medical places in the Fresno region (California). Three hierarchical levels are distinguished. First level: Fresno, Modesto, Bakersfield, second level: Merced, Visalia, and third level: Porterville, Hanford. We generated gravitational attractors for those centres, with the exception of Hanford, which lies too closely to the core city, Fresno. In this case the weight was the population number. The attractors obtained, and illustrated in Fig 7b, clearly show the structure of the Fresno medical care region as well as the spatial ranges of medical trade areas. Thus, they confirm Smith’s earlier result, but now obtained in a much more detailed and structured way, which demonstrates through an attractive visualization the functional hierarchical patterns in the area concerned.

## Scaling of the chaos-to-order transition derived from dimensional analysis

So far we have used arbitrary units, and therefore it is of considerable importance to rewrite Eq (10) in a reduced form:

$$\ddot{\tilde{\vec{r}}}=-\tilde{\mu }\dot{\tilde{\vec{r}}}-\sum_{i=1}^{M}\frac{\alpha_{i}}{{\tilde{r}}_{i}^{2}+{\tilde{h}}^{2}}\frac{{\tilde{\vec{r}}}_{i}}{\sqrt{{\tilde{r}}_{i}^{2}+{\tilde{h}}^{2}}},$$ (13)

where

$$\begin{array}{c} L=\sqrt{\frac{1}{M}\;\sum_{i=1}^{M}m_{i}{\left({\vec{r}}_{i}^{0}-{\vec{r}}_{M}\right)}^{2}}, \end{array}$$ (14)

$$\begin{array}{c} T=\frac{L^{3/2}}{\sqrt{k\bar{m}}}, \end{array}$$ (15)

$$\begin{array}{c} \bar{m}=\frac{1}{M}\sum_{i=1}^{M}m_{i}, \end{array}$$ (16)

$$\begin{array}{c} \tilde{\vec{r}}=\frac{\vec{r}}{L}, \end{array}$$ (17)

$$\begin{array}{c} \tilde{t}=\frac{t}{T}, \end{array}$$ (18)

$$\begin{array}{c} \tilde{\mu }=\mu \sqrt{\frac{L}{k\bar{m}}}, \end{array}$$ (19)

where ${\vec{r}}_{i}^{0}$ is the position of the *i*th city, and ${\vec{r}}_{M}$ is the position of the mass centre. Transition from chaotic to regular trajectories occurs approximately at ${\tilde{\mu }}_{c}$, which we estimate to be of the order of unity. Thus the true (unscaled) unit cost of transport which marks the onset of chaos, *μ*_*c*_, scales as:

$$\mu_{c}∼\sqrt{\frac{\bar{m}}{L}}.$$ (20)

**Fig 8:**
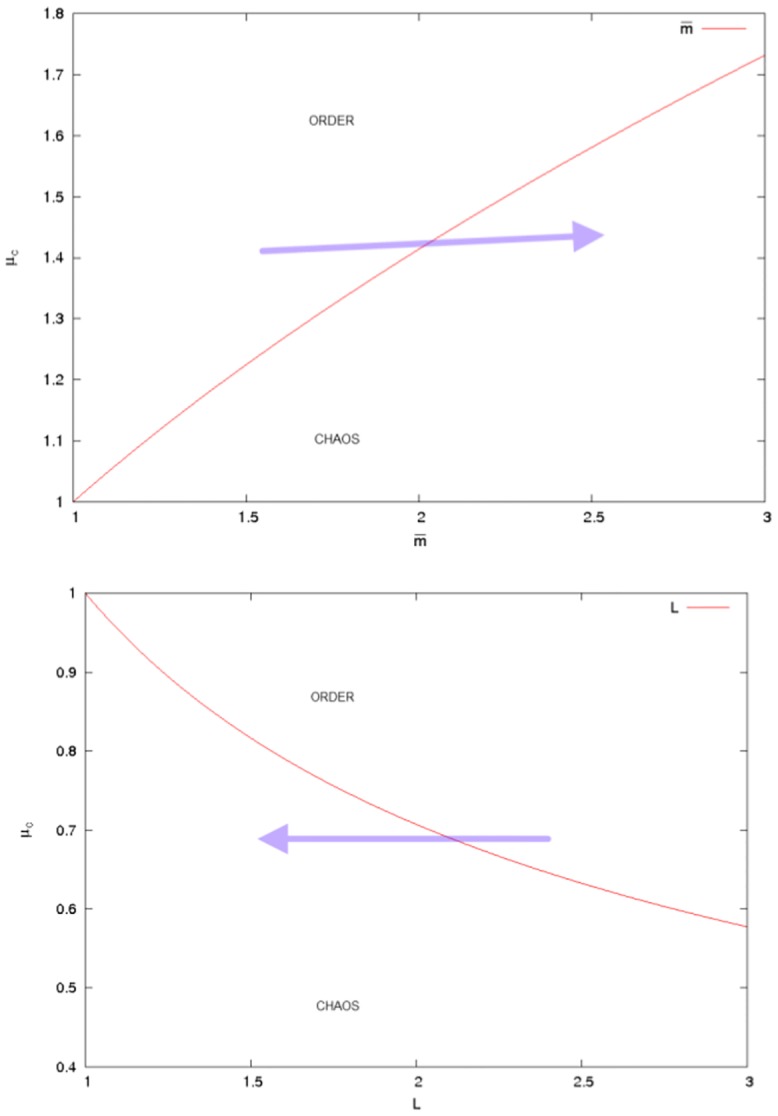
The scaling of *μ*_*c*_ corresponding to the onset of chaos with respect to *L* and $\bar{m}$.

We notice that the transition to chaos can be triggered either via increasing $\bar{m}$ or decreasing *L*, as indicated in Fig 8.

## Conclusions

The concept and identification of attraction basins—or attractors, to use the modern terminology—have been known since Newton’s times. However, it was only the discoveries by Mandelbrot, Hausdorff, Sierpinski and other influential physicists and mathematicians that have made this concept a universal research framework, especially within the framework of social physics and spatial sciences. In this paper the close relationship between gravitational impacts and spatial behaviour in socio-economic systems has been analysed. One of the factors that determines them is the resistance of space. This is a result of cost-minimizing spatial motions of interactive agents in geographical space. If the resistance is weak, spatial behaviour patterns assume a chaotic form. And vice versa, stronger resistance causes the system to stabilise by producing gravitational attractors, i.e. as a result of a phase transition. Then, another property is also revealed: when gravitational forces stabilise or disappear, the elements of the system interact via network linkages (see Nijkamp and Reggiani [15]). This finding corresponds to what CPT has foreseen. Advanced dynamic analyses of modern socio-economic systems corroborate this finding. In sum, the gravitational attractors discovered in our study are critical in explaining the properties of spatial impacts generated by urban units of various sizes. In conclusion, our analysis confirms also the validity of CPT in a dynamic space with interactive agents.

## Supporting Information

S1 Fig Results for a square. We enclose a fractal built on a square for *μ* = 0.08 when all vertices have the same mass (panel a). It is possible that the gravitational relationship between an agent and four cities represented by the fractal depends on the shape of the initial figure—a square in this case. Full spatial order for *μ* = 0.9 is presented in panel b. The square is divided into four attraction basins instead of six, when a hexagon is an initial figure at the beginning of the simulation process.
(TIFF)
