# Supplementary figures and images for: Self-Organisation in Spatial Systems—From Fractal Chaos to Regular Patterns and Vice Versa
Source: PLoS One. 2015 Sep 24;10(9):e0136248. doi: 10.1371/journal.pone.0136248 (PMC4581867; doi:10.1371/journal.pone.0136248)

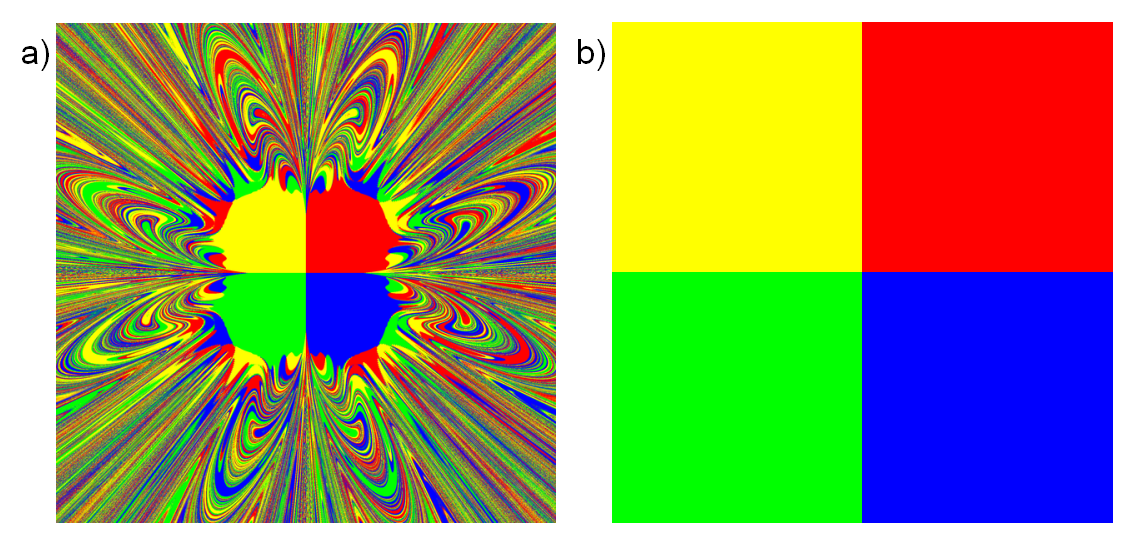

Supplement: S1 Fig — We enclose a fractal built on a square for μ = 0.08 when all vertices have the same mass (panel a). It is possible that the gravitational relationship between an agent and four cities represented by the fractal depends on the shape of the initial figure—a square in this case. Full spatial order for μ = 0.9 is presented in panel b. The square is divided into four attraction basins instead of six, when a hexagon is an initial figure at the beginning of the simulation process. (TIFF) [file pone.0136248.s001.tiff]
